# Exosomal release of the virus-encoded chemokine receptor US28 contributes to chemokine scavenging

**DOI:** 10.1016/j.isci.2023.107412

**Published:** 2023-07-18

**Authors:** Maarten P. Bebelman, Irfan M. Setiawan, Nick D. Bergkamp, Jeffrey R. van Senten, Caitrin Crudden, Jan Paul M. Bebelman, Frederik J. Verweij, Guillaume van Niel, Marco Siderius, D. Michiel Pegtel, Martine J. Smit

**Affiliations:** 1 Division of Medicinal Chemistry, Amsterdam Institute for Molecular and Life Sciences, Vrije Universiteit Amsterdam, de Boelelaan 1108, 1081 HZ Amsterdam, the Netherlands; 2 Department Pathology, Cancer Center Amsterdam, VU University Medical Center, de Boelelaan 1118, Amsterdam 1081 HZ, the Netherlands; 3 Division of Cell Biology, Neurobiology and Biophysics, Utrecht University, Padualaan 8, Utrecht 3584 CH, the Netherlands; 4 Institute of Psychiatry and Neuroscience of Paris (IPNP), INSERM U1266 Université de Paris, Paris, France

**Keywords:** Immunology, Cell biology

## Abstract

The human cytomegalovirus (HCMV)-encoded chemokine receptor US28 contributes to various aspects of the viral life cycle and promotes immune evasion by scavenging chemokines from the microenvironment of HCMV-infected cells. In contrast to the plasma membrane localization of most human chemokine receptors, US28 has a predominant intracellular localization. In this study, we used immunofluorescence and electron microscopy to determine the localization of US28 upon exogenous expression, as well as in HCMV-infected cells. We observed that US28 localizes to late endosomal compartments called multivesicular bodies (MVBs), where it is sorted in intraluminal vesicles. Live-cell total internal reflection fluorescence (TIRF) microscopy revealed that US28-containing MVBs can fuse with the plasma membrane, resulting in the secretion of US28 on exosomes. Exosomal US28 binds the chemokines CX_3_CL1 and CCL5, and US28-containing exosomes inhibited the CX_3_CL1-CX_3_CR1 signaling axis. These findings suggest that exosomal release of US28 contributes to chemokine scavenging and immune evasion by HCMV.

## Highlights

• US28 is sorted into the MVBs of human cytomegalovirus-infected cells
• Exocytosis of US28-containing MVBs results in the secretion of US28 on exosomes
• Exosomal US28 scavenges chemokines and inhibits human chemokine receptor activation

## Graphical abstract

**Figure:**
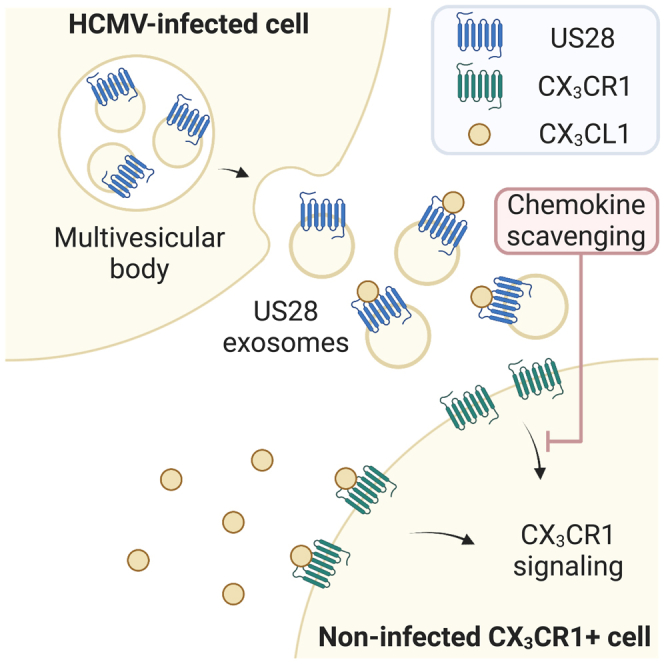

## Introduction

Human cytomegalovirus (HCMV) is a widespread human beta-herpesvirus with an estimated global seroprevalence of 83%.^1^ After primary infection, HCMV persists in the body in a latent state, and infection is generally asymptomatic in immune-competent individuals.^2^ However, reactivation in immunocompromised individuals, such as acquired immunodeficiency syndrome (AIDS) patients and organ transplant recipients, can lead to severe pathologies.^3^ Primary infection or reactivation from latency in pregnant women can result in congenital HCMV infection, a leading cause of neurological disease in children.^3^ Moreover, HCMV has been detected in various cancers, including glioblastoma, breast cancer, colon cancer, and prostate cancer, where it exhibits an oncomodulatory function.^4^

During the course of virus-host co-evolution, HCMV has acquired various host genes which have been modified to function in the viral life cycle. One of these captured genes is *US28*, which originates from the human chemokine receptor *CX3CR1* gene and encodes a viral G protein-coupled receptor (GPCR) with unique properties.^5^ Like its ancestor CX_3_CR1, the viral chemokine receptor US28 binds the chemokine CX_3_CL1, but it can also bind and signal in response to the CC-chemokines CCL5, CCL3, and CCL2.^6^^,^^7^ Furthermore, US28 constitutively activates a wide variety of signaling pathways depending on cellular context and stage of the viral infection cycle.^8^ US28 is expressed throughout the various stages of HCMV infection and contributes to a number of viral processes.^8^ Upon infection of CD14^+^ monocytes and CD34^+^ hematopoietic progenitor cells, constitutive US28 signaling represses transcription from the major immediate-early promoter (MIEP), which is crucial for the establishment and maintenance of HCMV latency.^9^^,^^10^^,^^11^ On the other hand, after the lytic phase in HCMV infection has been triggered, US28 signaling stimulates MIEP activation and contributes to cell-to-cell transmission in fibroblasts, epithelial cells, and vascular smooth muscle cells.^12^^,^^13^^,^^14^^,^^15^ In addition, CCL5- and CX_3_CL1-induced US28 signaling promotes the migration of vascular smooth muscle cells and macrophages, respectively.^16^ In HCMV-infected tumor cells, US28 activates several tumorigenic signaling pathways and thereby contributes to the oncomodulatory properties of HCMV.^17^

An interesting feature of US28 is its predominant intracellular localization, with only 20% of the receptor localized at the plasma membrane and the majority residing in intracellular compartments.^18^ This intracellular localization has been attributed to the constitutive endocytosis and recycling of US28, which results in removal of receptor-bound chemokines from the microenvironment of HCMV-infected cells.^19^^,^^20^^,^^21^ Chemokine scavenging by US28 has been demonstrated to neutralize CCL2- and CCL5-mediated migration of monocytes toward HCMV-infected fibroblasts, thereby contributing to immunosuppressive properties of the virus.^21^ Based on its scavenging role, one would expect US28 to primarily localize in early and recycling endosomes. However, electron microscopic analysis by Fraile-Ramos and colleagues revealed that US28 is highly enriched in the intraluminal vesicles (ILVs) of late endosomal compartments called multivesicular bodies (MVBs).^18^ When sorted into ILVs, US28 is presumably non-functional as it is physically separated from the cytosolic interactors that mediate downstream signaling. Furthermore, the fusion of US28-containing MVBs with lysosomes would result in substantial degradation of the receptor. This raises the question as to why it is beneficial for HCMV to express a receptor that is so heavily sorted toward this late endosomal compartment.

In the last decades it has become clear that not all MVBs are targeted for lysosomal degradation. Instead, a subset of MVBs can fuse with the plasma membrane, resulting in secretion of their ILVs as small extracellular vesicles (sEVs) called exosomes.^22^ Exosomes contain various cargo molecules, including mRNAs, microRNAs (miRNAs), and proteins, and play a role in intercellular communication by interacting with, or delivering cargo to, recipient cells.^23^ Many viruses, including herpesviruses, have been found to employ components of the host exosome biogenesis machinery for virion production.^24^^,^^25^ Furthermore, cells infected with the gamma-herpesviruses Epstein-Barr virus (EBV) and Kaposi sarcoma-associated herpesvirus (KSHV) have been found to secrete exosomes harboring viral miRNAs and proteins.^25^^,^^26^^,^^27^^,^^28^ In line with this, recent studies have reported that key drivers of exosome biogenesis contribute to HCMV virion production and that envelopment of HCMV virions occurs, in part, at MVB membranes.^29^^,^^30^^,^^31^ In addition, HCMV-infected cells release sEVs containing viral proteins, such as glycoprotein B (gB).^29^^,^^30^^,^^32^^,^^33^^,^^34^ Since US28 is mainly expressed intracellularly and highly enriched in MVBs, we hypothesized that US28 sorting into MVBs facilitates its secretion on exosomes and HCMV virions.

Here, we report that US28-containing MVBs can fuse with the plasma membrane, leading to the secretion of exosomes carrying US28. We further show that exosomal US28 retains the ability to bind the human chemokines CX_3_CL1 and CCL5 and that CX_3_CL1 scavenging by exosome-bound US28 modulates the CX_3_CL1-CX_3_CR1 signaling axis.

## Results

### US28 localizes to MVBs and is sorted into ILVs

**Figure 1:**
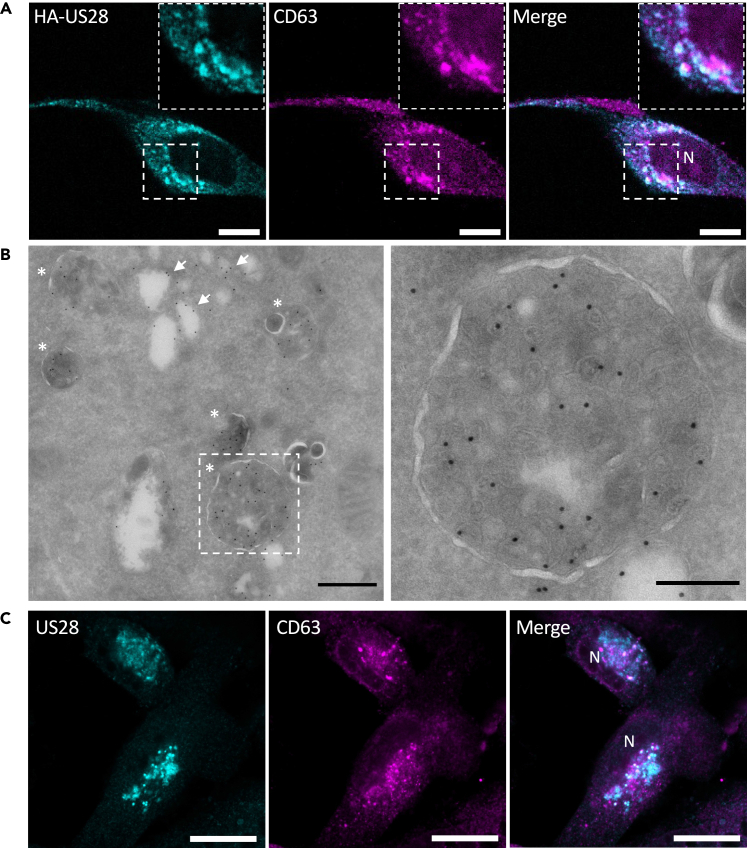
US28 is sorted into the ILVs of MVBs (A) Localization of HA-US28 (anti-HA staining) and the late endosomal marker CD63 in doxycycline-induced iHA-US28-U251 cells. Scale bar: 20 μm. N, nucleus. (B) Electron microscopic analysis of doxycycline-induced iHA-US28-U251 cells labeled with gold particles (10 nm) directed against HA-US28. Asterisks highlight US28-positive MVBs, and white arrows point at examples of US28 on other membranes. Scale bar left: 500 nm, right: 200 nm. (C) Localization of US28 (polyclonal anti-US28 antibody) and CD63 in HCMV Merlin-infected U251 cells 6 days post-infection. Scale bar: 20 μm. N, nucleus.

US28 localizes to intracellular compartments, both in HCMV-infected cells and when expressed exogenously in non-infected cells.^12^^,^^18^ Previous work demonstrated that US28 overexpression in HeLa cells results in its sorting into the ILVs of late endosomal compartments.^18^ Given the oncomodulatory role of US28 in glioblastoma and the intracellular expression of US28 in glioblastoma cells, we determined its localization in an iHA-US28-U251 glioblastoma cell line that allows for doxycycline-inducible hemagglutinin (HA)-tagged US28 expression.^35^ In line with previous findings,^18^^,^^36^ we observed partial colocalization between US28 and the late endosomal/lysosomal markers cluster of differentiation 63 (CD63) and lysosomal-associated membrane protein 1 (LAMP1) (Figures 1A and S1A). Furthermore, immunoelectron microscopy confirmed sorting of US28 into the ILVs of MVBs (Figure 1B). In addition, we observed US28 on intracellular membranes that appear to be early endosomes or enlarged Golgi tubules (Figure 1B). To determine US28 localization in the context of HCMV infection, we infected parental U251 cells with the clinical HCMV strain Merlin. At six days post-infection, the cells express US28, which partially co-localizes with CD63 as well as the cis-Golgi marker GM130 in a perinuclear compartment (Figures 1C and S1B). These findings confirm that US28 partially localizes to late endosomal compartments where it is sorted into ILVs.

### US28-containing MVBs can fuse with the plasma membrane to release exosomal US28

**Figure 2:**
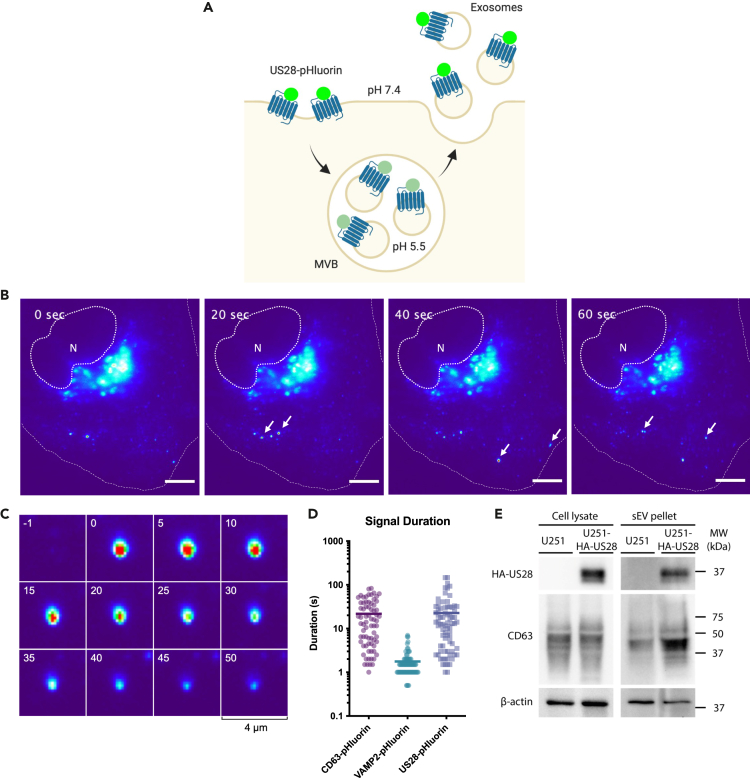
US28-containing MVBs fuse with the plasma membrane to release exosomal US28 (A) Schematic representation of US28-pHluorin-based visualization of MVB-plasma membrane fusion: the pH-sensitive pHluorin is quenched when facing the acidic lumen of the MVB. Upon fusion, the low luminal pH of the MVB is immediately neutralized, resulting in a sudden increase in fluorescence intensity. (B) TIRF imaging (heatmap) of a US28-pHluorin-expressing HeLa cell revealing the sudden appearance of multiple bright fluorescent spots at the plasma membrane. Stills corresponding to Video S1. New spots are highlighted by white arrows. N, nucleus. Dotted and dashed lines outline the nucleus and cell border, respectively. Scale bar: 10 μm. (C) Time-lapse imaging (heat maps) of a single fusion event of US28-pHluorin. (D) Fluorescence signal duration of individual CD63-pHluorin (n = 72, mean = 22 s), VAMP2-pHluorin (n = 79, mean 1.8 s), and US28-pHluorin (n = 76, mean = 23 s) fusion events. Graph depicts pooled data from 8 cells acquired in 2 independent experiments. (E) Western blot for HA-US28 (anti-HA antibody), the sEV marker protein CD63, and β-actin on cell lysates and the sEV pellet isolated by ultracentrifugation from equal numbers of U251 cells or doxycycline-induced iHA-US28-U251 cells. Representative of two independent experiments.

MVB maturation and fusion with lysosomes result in the degradation of its content. However, is has become clear that a subset of MVBs fuse with the plasma membrane instead, leading to secretion of the ILVs as exosomes.^22^ We previously developed a live-cell total internal reflection fluorescence (TIRF) microscopy approach using the reporter CD63-pHluorin that enables visualization of MVB-plasma membrane fusion.^37^^,^^38^ To determine whether US28-containing MVBs can fuse with the plasma membrane, we developed US28-pHluorin by inserting the pH-sensitive green fluorescent protein superecliptic pHluorin^39^ into the second extracellular loop of US28 (Figure 2A). US28-pHluorin retains functionality as it constitutively activates NFAT (Figures S2A and S2B) and localizes to CD63-positive endosomes (Figure S2C). We expressed US28-pHluorin in HeLa cells, which have a flat and spread-out plasma membrane that makes them ideal for studying exocytosis with TIRF microscopy.^37^ Upon live TIRF imaging of these cells, we detected multiple sudden localized bursts of fluorescence over time, suggesting fusion of US28-pHluorin-positive acidic compartments with the plasma membrane (Figures 2B and 2C, Video S1). We previously observed that MVB-plasma membrane fusion, as visualized by CD63-pHluorin, results in significantly longer signal duration when compared to plasma membrane deposition and lateral diffusion of vesicle-associated membrane protein 2 (VAMP2)-pHluorin.^38^ This prolonged fluorescence signal upon MVB-plasma membrane fusion most likely results from trapping of the released exosomes between the cells and the coverslips, preventing their fast diffusion. Thus, *signal duration* gives us an additional measure to differentiate exosome release from other exocytic events. For both US28- and CD63-pHluorin we observed several fusion events with a signal duration comparable to VAMP2-pHluorin (mean: 1.8 s), which likely result from plasma membrane deposition of US28 and CD63 by recycling endosomes or transport vesicles (Figure 2D). However, the signal duration of the majority of US28-pHluorin fusion events was longer (mean: 23 s) and similar to the duration of CD63-pHluorin fusion events (mean: 22 s), suggesting that most fusion events result from the fusion of US28-pHluorin-containing MVBs with the plasma membrane. In line with this, we could detect US28 in the sEV pellet from the supernatant of doxycycline-induced iHA-US28 U251 cells (Figure 2E).

Video S1. US28-containing MVBs fuse with the plasma membrane, related to Figure 2 TIRF imaging (heatmap) of a US28-pHluorin-expressing HeLa cell revealing the sudden appearance of multiple bright fluorescent spots at the plasma membrane. Displayed at 10x normal speed. Scale bar: 10 μm.

### HCMV-infected cells secrete US28 on small EVs and virions

**Figure 3:**
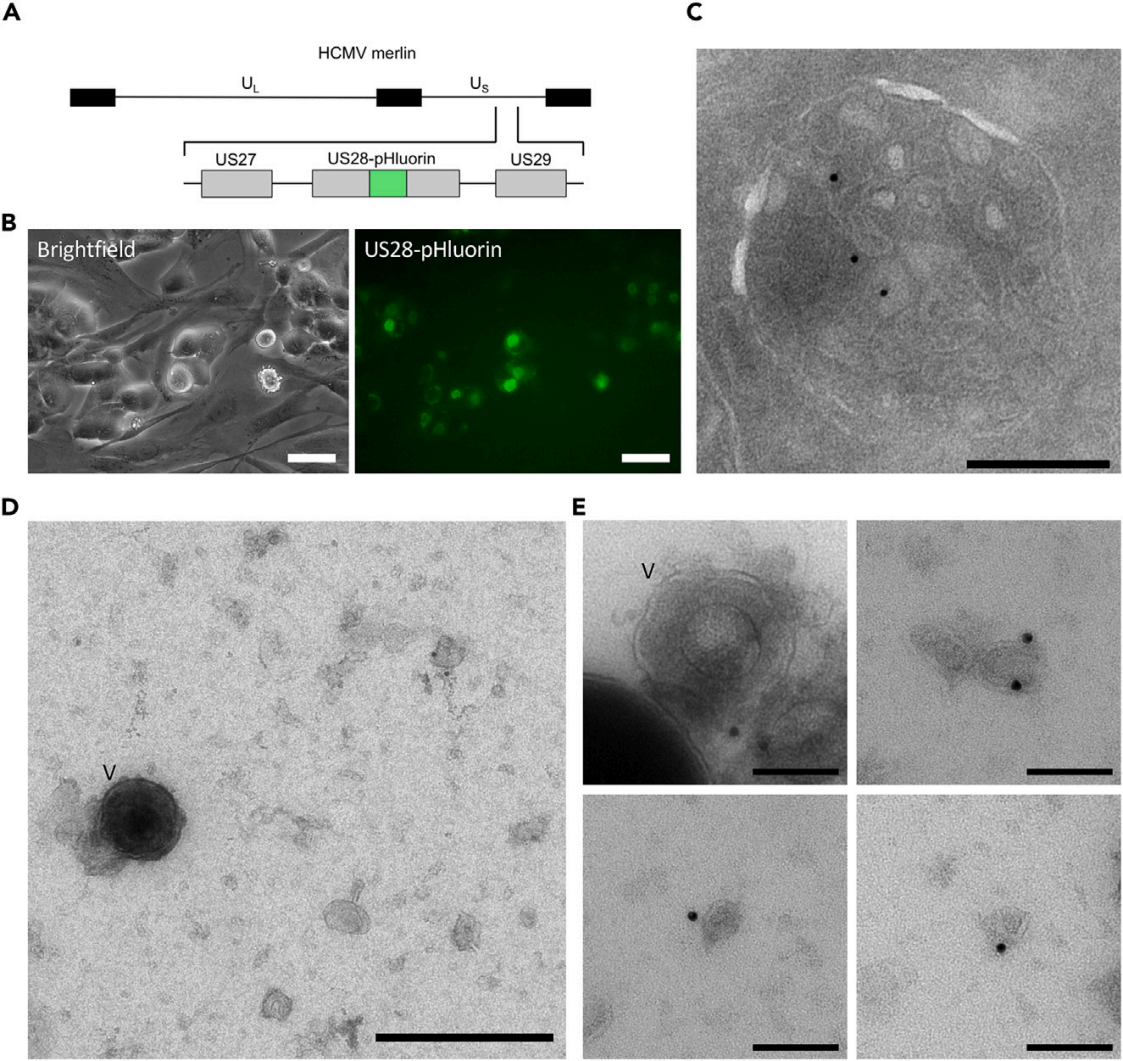
US28 is present on sEVs and virions from HCMV-infected fibroblasts (A) Schematic representation of the insertion of pHluorin in the 2^nd^ extracellular loop of HCMV Merlin US28. (B) Bright-field (left) and fluorescence microscopy (right) of HCMV-US28-pHluorin-infected HFFF-Tet cells. Green fluorescence signal can be observed in the perinuclear VAC of cells showing cytopathic effects. Scale bars: 50 μm. (C) Electron microscopic analysis of HCMV-US28-pHluorin infected HFFF-Tet cells labeled with gold particles (10 nm) directed against US28-pHluorin (anti-GFP). Scale bar: 250 nm. (D and E) Electron microscopic analysis of mature virions and sEVs in the supernatant of HCMV-US28-pHluorin infected HFFF-Tet cells with gold labeling against US28-pHluorin (anti-GFP). Mature virions are highlighted with a V. Scale bars: 250 nm (D) and 100 nm (E).

To study exosome-mediated secretion of US28 in the context of HCMV infection, we used bacterial artificial chromosome-based recombineering to generate HCMV-US28-pHluorin from the low-passage HCMV strain Merlin^40^ (Figure 3A). In HCMV-US28-pHluorin-infected human fetal foreskin fibroblasts (HFFF-Tet cells^40^), green fluorescent signal could be observed in the perinuclear virion assembly compartment (VAC) (Figure 3B), indicating the presence of US28-phluorin in non-acidic compartments in the VAC, such as the Golgi. To assess whether US28-pHluorin also localizes to MVBs in infected fibroblasts, we performed immunoelectron microscopy using an anti-GFP-antibody that recognizes pHluorin. Similar to our observations in cells overexpressing US28, we could detect gold labeling on the small ILVs within MVBs (Figure 3C). Unfortunately, the high background caused by US28-pHluorin in non-acidic compartments in the VAC hampered the live TIRF imaging of MVB-plasma membrane fusion events in HCMV-infected cells. To investigate the presence of US28 in EVs secreted by HCMV-infected cells, we used ultracentrifugation to pellet both HCMV virions and sEVs from the supernatant of HCMV-infected fibroblasts and performed immunoelectron microscopy using anti-GFP-gold labeling (Figures 3D and 3E). Consistent with previous findings,^9^ we did occasionally observe gold labeling on HCMV virions, which could be identified by their size of ±200 nm and the presence of a viral capsid^41^ (Figure 3E, upper left panel). In addition, we observed the association of gold particles with 100–150 nm sEVs, corresponding to the size of MVB-derived exosomes (Figures 3D and 3E). These results demonstrate that HCMV-infected cells release US28 both on mature virions and on sEVs.

### Exosomal US28 scavenges chemokines and modulates human chemokine receptor activation

**Figure 4:**
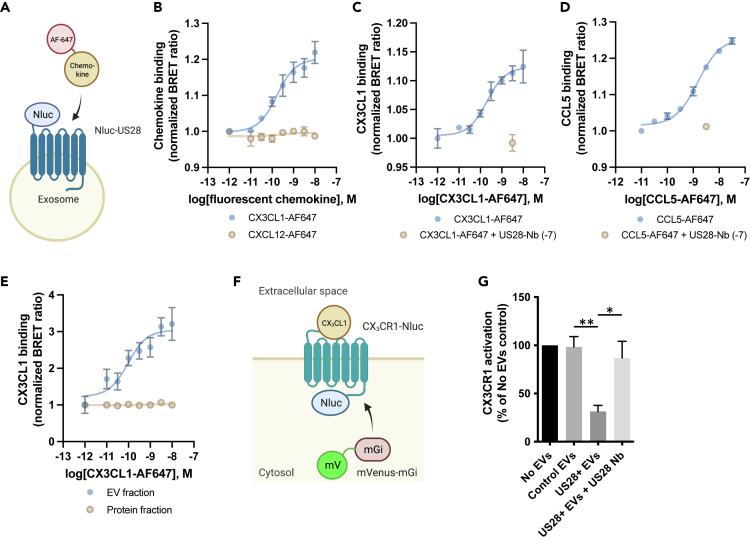
Exosomal US28 scavenges chemokines and modulates CX_3_CR1 activation (A) Schematic representation of the NanoBRET approach to measure chemokine binding to EV-bound NanoLuc-US28 (Nluc-US28). (B) Binding of increasing concentrations of CX_3_CL1-AlexaFluor-647 or CXCL12-AlexaFluor-647 to EV-bound Nluc-US28. Representative graph of three independent experiments is shown with the mean ± SD of triplicate values. (C and D) Binding of increasing concentrations of CX_3_CL1-AlexaFluor-647 (C) or CCL5-AlexaFluor-647 (D) to EV-bound Nluc-US28, with or without addition of anti-US28 nanobody (US28-Nb) (VUN100, 100 nM). Representative graphs of three independent experiments are shown with the mean ± SD of triplicate values. (E) Binding of increasing concentrations of CX_3_CL1-AlexaFluor-647 to Nluc-US28 in the EV and protein fractions after size-exclusion chromatography. Representative graph of three independent experiments is shown with the mean ± SD of triplicate values. (F) Schematic representation of the assay setup to measure CX_3_CR1 activity. Activation of Nluc-tagged CX_3_CR1 results in recruitment of mVenus-Mini-G_i_ and a concomitant increase in BRET signal. (G) CX_3_CR1 activation in HEK293 cells upon stimulation with CX_3_CL1 (1 nM), in the presence of control or US28-containing EVs, with or without anti-US28 nanobody (VUN100, 31.6 nM). Graph depicts the mean ± SEM of three independent experiments with three replicates per experiment. For each condition, the increase in BRET upon CX3CL1 stimulation was presented as a percentage of the increase in BRET upon CX_3_CL1 in the absence of EVs (depicted as 100% CX_3_CR1 activation). ∗, p < 0.05; ∗∗, p < 0.01. Statistical analyses were done using Student’s two-tailed two-sample t test.

The secretion of US28 on exosomes raises the question as to what the functional relevance of this process during HCMV infection could be. It has been reported that mesenchymal stem cell EVs containing the chemokine receptor CCR2 act as chemokine scavengers.^42^ Given the proposed scavenging function of US28, we set out to investigate whether exosomal US28 modulates the activity of extracellular chemokines. First, we determined whether exosomal US28 can indeed bind to its ligands CX_3_CL1 and CCL5. For this, we used a NanoBRET approach to measure the binding of fluorescently labeled chemokines to exosomes containing NanoLuc (Nluc)-tagged US28 (Figure 4A). Exposure of supernatant from Nluc-US28-transfected HEK293T cells to fluorescent CX_3_CL1 or CCL5 resulted in a concentration-dependent increase in bioluminescence resonance energy transfer (BRET) signal, whereas fluorescent CXCL12, which is not a ligand for US28, did not increase the BRET signal (Figures 4B–4D). Importantly, the increase in BRET signal obtained with fluorescent CX_3_CL1 or CCL5 could be counteracted by competition with a saturating concentration of anti-US28 nanobody (VUN100), which has previously been demonstrated to displace CX_3_CL1 and CCL5 from US28^43^ (Figures 4C and 4D). To ensure that we specifically measured chemokine binding to exosome-associated Nluc-US28, we repeated the NanoBRET assay after separation of EVs and soluble proteins from the culture supernatant by size-exclusion chromatography (SEC) and could only observe an increase in BRET signal for the EV fraction (Figure 4E). These findings show that the increase in BRET is caused by specific interactions of fluorescent CX_3_CL1 and CCL5 with exosomal US28 and demonstrate that exosomal US28 has the potential to scavenge chemokines.

To investigate whether chemokine scavenging by exosomal US28 could influence human chemokine receptor signaling, we determined the effect of exosomal US28 on CX_3_CL1-induced activation of CX_3_CR1. CX_3_CR1 activation was measured using a BRET-based assay that measures recruitment of Mini G_i_ proteins^44^ to CX_3_CR1-Nluc (Figure 4F). US28-containing EVs were isolated from the culture supernatant of doxycycline-stimulated iHA-US28-U251. In the presence of US28-containing EVs, but not US28-negative control EVs, CX_3_CL1-induced recruitment of mini Gα_i_ proteins to CX_3_CR1 was inhibited by over 60% (Figure 4G). Moreover, preincubation of US28-containing EVs with the anti-US28 nanobody VUN100 rescued CX_3_CR1 activation, showing that this inhibitory effect is indeed US28 dependent. Together these results suggest that exosomal US28 can modulate human chemokine receptor signaling by scavenging soluble chemokines.

## Discussion

The viral chemokine receptor US28 is a versatile receptor that contributes to various aspects of HCMV infection, such as chemokine scavenging, cell-to-cell transmission, and the establishment of latency.^8^ In contrast to the plasma membrane localization of most human chemokine receptors, the majority of US28 is localized within the ILVs of MVBs.^18^ Sorting of US28 into MVBs has been shown to be mediated by the adapter GPCR-associated binding protein 1 (GASP1),^36^ which facilitates the incorporation of GPCRs into ILVs by linking them to the endosomal sorting complexes required for transport (ESCRT) protein hepatocyte growth factor regulated tyrosine kinase substrate (HRS).^45^ The sorting of US28 into ILVs has been suggested to lead to its degradation in lysosomes, but the reason for such extensive degradation was unclear. In this study, we show that alternatively to fusion with lysosomes, a subset of the US28-containing MVBs can fuse with the plasma membrane and release US28-containing ILVs into the extracellular space as exosomes.

US28 sorting into ILVs and its subsequent secretion on exosomes might serve a number of different purposes that could be beneficial for HCMV. Firstly, US28 is constitutively active and in recent years it has become clear that activated GPCRs can sustain signaling from endosomal compartments.^46^ This limits the possibilities for HCMV to control US28 signaling in infected cells and increases the risk of overactivation of US28 signaling pathways, which could be detrimental for the host cell and have undesirable consequences on viral persistence. The sorting of US28 into ILVs and its subsequent release via exosomes or degradation in lysosomes could help to regulate US28 signaling. In fact, another herpesvirus, the Epstein-Barr virus (EBV), employs a similar strategy to control constitutive nuclear factor κB (NF-κB) activation by the viral latent membrane protein-1.^47^ Secondly, sorting of US28 to MVBs could be a strategy to enable efficient incorporation of US28 into the viral envelope during virion maturation. Although for a long time HCMV envelopment was believed to occur mostly at early endosome and Golgi membranes,^48^^,^^49^ a growing body of evidence suggests that the host exosome biogenesis pathway underlies virion production^29^^,^^30^ and that HCMV envelopment at least partially occurs at the MVB limiting membrane.^31^ HCMV infection causes remodeling of the secretory pathway, in part through viral miRNAs that target members of the endocytic pathway, such as ras-related protein rab-5 (RAB5) and synaptosomal-associated protein 23 (SNAP23), which could influence MVB exocytosis dynamics in infected cells.^50^ However, multi-color TIRF microscopy using CD63-pHluorin, the viral glycoprotein gM/UL100-mScarlet-I, and the tegument protein pp150-SNAP revealed that virion-containing MVBs can fuse with the plasma membrane.^31^ A potential reason for the sorting of US28 into virions became apparent, when direct insertion of US28 from the viral envelope into the plasma membrane of newly infected cells was found to contribute to the establishment of latency.^9^^,^^10^ The direct delivery of US28 protein mediates the suppression of the MIEP immediately after infection until new US28 is synthesized by the infected cell.^10^

Is the presence of US28 in exosomes solely a consequence of an attempt to regulate signaling or sorting into the viral envelope, or does exosomal US28 itself play a role during HCMV infection? In this study, we showed that exosomal US28 is capable of binding the chemokines CX_3_CL1 and CCL5, reducing the concentrations of free chemokines and thus leading to reduced cellular chemokine receptor activation. Notably, a previous study reported that mesenchymal stem cell EVs containing the chemokine receptor CCR2 act as chemokine scavengers that suppress CCL2-induced macrophage migration and activation,^42^ suggesting that chemokine scavenging by EV-associated chemokine receptors is a more common feature of chemokine receptors. In the context of HCMV infection, exosomal US28 could reduce chemokine-dependent chemotaxis of immune cells toward the site of infection. In addition to chemokine scavenging by constitutive endocytosis and recycling of cellular US28, the secretion of US28 on exosomes may enable HCMV to disrupt chemokine gradients further away from the infection site. As such, the secretion of US28-containing exosomes could help HCMV-infected cells to escape immune surveillance. Similarly, chemokine scavenging by various human chemokine receptors that are present in cancer cell-derived EVs^51^^,^^52^ may have significant implications for anti-tumor immune responses. Interestingly, two other HCMV-encoded GPCRs, UL33 and US27, also sorted into ILVs,^53^ have recently been identified in density-gradient ultracentrifugation-purified sEVs from the supernatant of HCMV-infected cells.^30^ Unlike US28, these receptors are not known to bind chemokines, indicating that chemokine scavenging might not be the only reason for EV-mediated secretion of viral GPCRs.

Besides their interaction with extracellular matrix components, exosomes and other EVs mediate intercellular communication by delivering their cargo to recipient cells. Exosome-mediated transfer of viral material is increasingly recognized as a mechanism by which herpesviruses manipulate non-infected cells in their environment,^25^ and HCMV exosomes have been shown to promote viral spread in fibroblasts.^32^ EV-mediated transfer of functional receptors has been described for various GPCRs, including the human chemokine receptors CXCR4^52^^,^^54^^,^^55^ and CCR5.^56^ Whether exosomal US28 can be functionally transferred to non-infected cells remains to be investigated. However, given the role of virion-associated US28 in the establishment of latency, it would be of interest to see whether functional delivery of exosomal US28 contributes to the immediate MIEP silencing observed at early time points after infection of monocytes and hematopoietic progenitor cells.

Over the years, it has become clear that the viral GPCR US28 regulates a wide variety of viral processes throughout the various stages of HCMV infection.^8^ In this study, we demonstrated that the functionality of US28 extends beyond its roles in infected cells or in virions; exosome-associated US28 contributes to chemokine scavenging. These findings provide an explanation for the localization of US28 in MVBs and pave the way to further explore additional functionalities of exosome-mediated viral GPCR secretion by HCMV.

### Limitations of the study

In this study we demonstrated that US28-containing MVBs can fuse with the plasma membrane to release US28 on exosomes. Furthermore, we observed US28 within MVBs and in the sEV pellet of HCMV-infected cells. However, the presence of US28-pHluorin in non-acidic compartments hampered the live TIRF imaging of MVB exocytosis in HCMV-US28-pHluorin-infected cells. Therefore, we cannot exclude that (a fraction of) the US28-containing sEVs from HCMV-infected cells bud directly from the plasma membrane. Importantly, the size of the US28-containing sEVs is consistent with an endosomal origin and our findings are consistent with a recent study that demonstrated MVB-plasma membrane fusion in HCMV-infected cells using CD63-pHluorin.^31^

While it is clear that chemokine scavenging by exosomal US28 inhibits CX_3_CR1 signaling, further studies should be performed to assess the effect of chemokine scavenging by exosomal US28 on immune evasion by HCMV-infected cells. Similarly, the potential horizontal transfer of exosomal US28 to non-infected recipient cells warrants further investigation as this could have implications for the establishment of latent HCMV infection.

## STAR★Methods

### Key resources table

**Table:** 

REAGENT or RESOURCE	SOURCE	IDENTIFIER
**Antibodies**		

Rabbit anti-US28	M.J. Smit (VU University Amsterdam, The Netherlands) Heukers et al.^59^	N/A
Rat anti-HA	Roche	cat. #: 11867423001; RRID:AB_390918
Rabbit anti-HA	Abcam	cat. #: ab9110; RRID:AB_307019
Mouse anti-CD63	BD Biosciences	cat. #: 556019; RRID:AB_396297
Rabbit anti-LAMP1	Cell Signaling	cat. #: 9091; RRID:AB_2687579
Mouse anti-GM130	BD Biosciences	cat. #: 610822; RRID:AB_398141
Goat Alexa Fluor®594-linked anti-mouse secondary antibody	Thermo Fisher Scientific	cat. #: A-11032; RRID:AB_2534091
Goat Alexa Fluor®488-linked anti-rat secondary antibody	Thermo Fisher Scientific	cat. #: A-11006; RRID:AB_2534074
Goat Alexa Fluor®594-linked anti-rabbit secondary antibody	Thermo Fisher Scientific	cat. #: A-11012; RRID:AB_2534079
Goat Alexa Fluor®488-linked anti-rabbit antibody	Thermo Fisher Scientific	cat. #: A-11008; RRID:AB_143165
Rabbit anti-GFP	Thermo Fisher Scientific	cat. #: A-11122; RRID:AB_221569
Mouse anti-β-actin	Sigma Aldrich	cat. #: A5316; RRID:AB_476743
Goat anti-rat horseradish peroxidase (HRP)-conjugated antibody	Thermo Fisher Scientific	cat. #: 31470; RRID:AB_228356
Goat anti-mouse-HRP-conjugated antibody	Bio-Rad	cat. #: 170-6516; RRID:AB_11125547

**Bacterial and virus strains**		

SW102 E. coli carrying the HCMV Merlin BAC pAL1502	R.J. Stanton (Cardiff University, Cardiff, UK) Stanton et al.^40^	N/A
HCMV Merlin US28-pHluorin	This study	N/A

**Chemicals, peptides, and recombinant proteins**		

Amaxa basic fibroblast nucleofector kit	Lonza	Cat. #: VPI-1002
NanoGlo Luciferase Assay System	Promega	Cat. #: N1110
Monovalent US28 nanobody VUN100	M.J. Smit (VU University Amsterdam, The Netherlands) De Groof et al.^43^	N/A
CX_3_CL1-Alexa647	Almac Group	Cat. #: CAF-14
CCL5-Alexa647	Almac Group	Cat. #: CAF-8
CXLC12-Alexa647	Almac Group	Cat. #: CAF-11

**Experimental models: Cell lines**		

HFFF-Tet	R.J. Stanton (Cardiff University, Cardiff, UK) Stanton et al.^40^	N/A
iHA-US28-U251	M.J. Smit (VU University Amsterdam, The Netherlands) van Senten et al.^35^	N/A
HEK293T	ATCC	Cat. #: CRL-3216; RRID:CVCL_0063
HeLa	Sigma Aldrich	ECACC, cat. #: 93021013; RRID:CVCL_0030

**Recombinant DNA**		

HA-US28-pHluorin in pcDEF3	This study	N/A
HA-US28-pHluorin in pCMV-SPORT6	This study	N/A
Nluc-US28 in pcDEF3	M.J. Smit (VU University Amsterdam, The Netherlands) De Groof et al.^57^	N/A
VAMP2-pHluorin in pCI	J. Rothman (Yale University, New Haven, CT)	N/A
CD63-pHluorin in pCMV-SPORT6	D.M. Pegtel (Amsterdam UMC, The Netherlands) Verweij et al.^38^	N/A
CD63-pHuji in pCMV-SPORT6	D.M. Pegtel (Amsterdam UMC, The Netherlands) Verweij et al.^38^	N/A
3xHA-CX_3_CR1 in pcDNA3.1	cDNA Resource Center (Bloomsburg, USA)	Cat. #: CX3R10TN00
3xHA-CX_3_CR1-Nluc in pcDEF3	This study	N/A
NES-Venus-mGsi143 in pcDEF3	This study	N/A
HA-US28 in pcDEF3	M.J. Smit (VU University Amsterdam, The Netherlands)	N/A

**Software and algorithms**		

FIJI	Schindelin et al.^60^	N/A
Prism 9.0 software	GraphPad	N/A

### Resource availability

#### Lead contact

Further information and requests for resources and reagents should be directed to and will be fulfilled by the lead contact, Martine J. Smit (mj.smit@vu.nl).

#### Materials availability

Plasmids and recombinant HCMV strains generated in this study will be made available upon request. A material transfer agreement will be required prior to sharing of materials.

### Experimental model and study participant details

#### Cell lines

HFFF-Tet cells were kindly provided by Dr. Richard J. Stanton.^40^ HFFF-Tet cells, HEK293T, iHA-US28-U251^35^ and HeLa cells were cultured in a humidified atmosphere with 5% CO_2_ at 37°C in DMEM (Gibco), supplemented with 10% fetal bovine serum (FBS; Gibco) and 50 IU/mL penicillin and streptomycin (PAA). HA-US28 expression in iHA-US28-U251 cells was induced using 1 μg/mL doxycycline (D9891, Sigma-Aldrich).

### Method details

#### Plasmids

To generate HA-US28-pHluorin, Site-directed mutagenesis with primers 5′-*gaccgactacgactacagatctttagaggtcagttacc*-3′ and 5′-*ggtaactgacctctaaagatctgtagtcgtagtcggtc*-3′ was used to introduce a BglII restriction site between Y179 and L180 in the second extracellular loop of HA-US28-pcDEF3. pHluorin was obtained by BglII digestion of CD63-pHluorin-pCMV-sport6^38^ and ligated into BglII-digested HA-US28. For TIRF experiments, HA-US28-pHluorin was subcloned into pCMV-sport6 using EcoRI and XbaI restriction sites. The pcDEF3 vector containing Nluc-US28 was described previously.^57^ The VAMP2-pHluorin was a gift from J. Rothman (Yale University, New Haven, CT), generation of the CD63-pHluorin plasmid was described previously.^38^ The 3xHA-CX_3_CR1 pcDNA3.1 plasmid was ordered from the cDNA Resource Center (Bloomsburg, USA). To generate the 3xHA-CX_3_CR1-Nluc plasmid, 3xHA-CX_3_CR1 was PCR-amplified using the following primers: Fw - 5′ GAAATTAATACGACTCACTATAGGG 3′ and Rv: 5′ ATAGCGGCCGCGAGAAGGAGCAATGCATCTCC 3’. Next, the PCR-amplified 3xHA-CX_3_CR1 was put in frame at the N-terminus of Nluc in the pcDEF3 vector. The previously described NES-Venus-mGsi143 (Venus-mini-Gαi) construct^44^ was ordered from Twist Bioscience (San Francisco, California, United States) and subcloned to the pcDEF3 vector.

#### NFAT reporter gene assay

HEK293T cells in suspension were co-transfected with pNFAT-FLuc (Firefly Luciferase reporter) and equal amounts of HA-US28 wildtype, HA-US28-pHluorin or empty pcDEF_3_ vector (Mock) using Linear polyethylenimine (PEI) (Polysciences) and subsequently plated in white flat-bottomed 96-well plates. Luciferase activity was measured 24h post-transfection using a Victor3 multilabel plate reader (PerkinElmer Life Sciences).

#### Enzyme-linked Immunosorbent assay (ELISA)

Transfected cells from the NFAT reporter gene assay were seeded in poly-L-lysine-coated transparent 96-well plates. After 24 h, cells were fixed with 4% paraformaldehyde (Sigma-Aldrich) for 10 min at room temperature (RT), permeabilized with 0.5% NP-40 (Sigma-Aldrich) for 20 min at RT and subsequently blocked using 2% skimmed milk powder in PBS for 1 h at RT. Cells were incubated with anti-HA antibody (rat, clone 3F10, Roche) for 1 h at RT. Subsequently, cells were washed with PBS and then incubated with goat anti-Rat IgG-HRP conjugate (Pierce, Thermo Scientific) for 1 h at RT. After washing with PBS, 1-Step Turbo TMB-ELISA substrate (Thermo Scientific) was added to the wells and the reaction was stopped with 1 M H_2_SO_4_. Optical density was measured at 450 nm using a PowerWave plate reader (Biotek).

#### HCMV merlin BAC recombineering

SW102 E. coli carrying the HCMV Merlin BAC pAL1502, a variant of BAC pAL1498^40^ lacking the eGFP tag, were acquired from Dr. Richard J. Stanton. Recombineering was performed using galK positive/negative selection as previously described by Warming et al.^58^ Adaptations to the protocol used to generate the HCMV-US28-pHluorin Merlin BAC recombinant are specified below. The galK expression cassette was amplified from pgalK (Fredrick National Laboratory for Cancer Research) by PCR using oligonucleotide primers with homology arms flanking the pHluorin insertion site between Y179 and L180 of the US28: 5′-*ttatggtggtgaccaaaaaagacaatcaatgtatgaccgactacgactaccctgttgacaattaatcatcggca*-3′ and 5′-*gcaccgagcatgagttctacgttgaggatgatcgggtaactgacctctaatcagcactgtcctgctcctt*-3’.

To remove galK and generate HCMV-US28-pHluorin, pHluorin was amplified by PCR using oligonucleotide primers with homology arms flanking the insertion site: 5′-*ttatggtggtgaccaaaaaagacaatcaatgtatgaccgactacgactacagatctctagccaccatgggaag*-3′ and 5′- *gcaccgagcatgagttctacgttgaggatgatcgggtaactgacctctaaagatctgattcgagctccaccg*-3’. BAC DNA was isolated using NucleoBond Xtra BAC kit (Machery Nagel).

#### Virus production

HFFF-Tet cells were transfected with HCMV-US28-pHluorin BAC DNA (2 μg per 2x10^6^ cells) using the Amaxa basic fibroblast nucleofector kit (Lonza) and an Amaxa Nucleofector (Lonza). Subsequent virus productions were initiated by infection of HFFF-Tet cells at MOI 0.02. Expression of RL13 and the UL128 locus were repressed during virus production.

#### Immunofluorescence microscopy

Cells were fixed using 4% PFA (20 min, RT), blocked and permeabilized in PBS containing 0.05% saponin and 2% BSA (1 h, rt), and subsequently stained in blocking solution (1h, rt) using antibodies against US28 (rabbit polyclonal, Covance),^59^ HA-tag (rat, cat. #: 11867423001, Roche) CD63 (mouse, cat. #: 556019, BD Biosciences), LAMP1 (rabbit, cat. #9091, Cell Signaling) and GM130 (mouse, cat. #: 610822, BD biosciences). Alexa Fluor® 594-linked anti-mouse secondary antibody (cat. #: A-11032, Thermo Fisher Scientific), Alexa Fluor® 488-linked anti-rat secondary antibody (cat. #: A-11006, Thermo Fisher Scientific), Alexa Fluor® 594-linked anti-rabbit secondary antibody (cat. #: A-11012, Thermo Fisher Scientific), and Alexa Fluor® 488-linked anti-rabbit antibody (cat. #: A-11008, Thermo Fisher Scientific) were used as secondary antibodies. Cell nuclei were stained using 4′,6-diamidino-2-phenylindole (DAPI) (D9542, Sigma-Aldrich).

Confocal laser scanning microscopy was performed using a Nikon A1R+ microscope (Nikon, Tokyo, Japan) equipped with a 60 ×1.4 oil-immersion objective or a 100×1.45 oil-immersion objective, and a Leica DMRB microscope (Leica, Cambridge, UK) equipped with a 40×1.00 oil-immersion objective. NIS-Elements (Nikon, Tokyo, Japan) and Leica Confocal Software (Leica, Cambridge, UK) were used for image acquisition and Fiji^60^ was used for image analysis.

#### Immuno-electron microscopy

Doxycyclin-induced iHA-US28-U251 cells and HCMV-US28-pHluorin-infected HFFF-Tet cells were fixed in 2% PFA, 0.2% glutaraldehyde in 0.1M phosphate buffer pH 7.4. Cells were then washed with phosphate buffer, embedded in 10% (wt/vol) gelatin and infused in 2.3 M sucrose. Mounted gelatin blocks were frozen in liquid nitrogen and ultrathin sections were prepared with an Ultracut FCS ultracryomicrotome (Leica). Ultrathin cryosections were labeled with rabbit anti-HA (cat. #: ab9110, Abcam, 1:500) or rabbit anti-GFP (cat. #: A11122, Invitrogen, 1:200) antibodies, and protein A coupled to 10nm gold particles. EVs and virions were isolated from the culture medium of HCMV-infected HFFF-Tet cells. Culture medium was centrifuged at 500xg to remove dead cells and cell debris and subsequently centrifuged for 1 hour at 23,000 rpm to isolate EVs and virions. EVs and virions were spotted on carbon-coated and formvar coated EM grids and fixed with 2% PFA in PBS before staining with anti-GFP (antibody and protein A coupled to 10nm gold particles. Samples were examined with a FEI Tecnai Spirit electron microscope (FEI Company), and digital acquisitions were made with a numeric camera (Quemesa; Soft Imaging System).

#### TIRF microscopy

For US28-pHluorin TIRF imaging, HeLa cells on poly-l-lysine-coated round 18 mm coverslips in a 12 wells plate were transfected with 500 ng HA-US28-pHluorin plasmid using Lipofectamine 2000 (Invitrogen). 24 hours after transfection, coverslips were placed in an imaging chamber, perfused with Tyrode’s solution and imaged on a microscope (Zeiss, Axiovert 200M) equipped with an EMCCD camera (Cascade, Roper Scientific). For TIRF imaging, a laser beam from an air-cooled argon ion laser was coupled into a 100 × 1.45 N.A. TIRF objective via a TIRF condenser (TILL Photonics). Images were acquired at 2 Hz with MetaMorph 6.2 software (Universal Imaging). Imaging experiments were performed at room temperature (21°C–24°C).

#### sEV isolation for western blot

For western blot on the sEV pellet, culture supernatant from doxycycline-induced and control iHA-US28-U251 cells was collected and sequentially centrifuged at 500xg for 20min, 2000xg for 20min, and 10,000xg for 30min to remove dead cells and cell debris. EVs were isolated by ultracentrifugation at 120,000xg for 70min using a Beckman-Coulter Optima XE-90 ultracentrifuge and SW32ti rotor (Fullerton, CA).

#### Western blot

Isolated EVs were run on a 10% SDS gel and blotted on a PVDF membrane. Membranes were probed with antibodies against HA (rat, cat. #: 11867423001, Sigma-Aldrich), CD63 (mouse, cat. #: 556019, BD Biosciences) and β-actin (mouse, cat. #: A5316, Sigma-Aldrich). Secondary antibodies used were anti-rat horseradish peroxidase (HRP)-conjugated antibody (cat. #: 31470, Thermo Fisher Scientific) or anti-mouse-HRP-conjugated antibody (cat. #: 170-6516, Bio-Rad). Protein expression was visualized using ECL substrate (32209; Pierce) and a ChemiDoc™ MP Imaging System (Bio-Rad).

#### Fluorescently labeled chemokine binding assay

To collect Nluc-US28-expressing EVs, two million HEK293T cells were seeded in a 10cm^2^ dish (Greiner Bio-one). The next day, cells were transfected with 250 ng of Nluc-US28 supplemented with empty pcDEF3 vector to total of 5 μg DNA and 30μg Linear polyethylenimine (PEI) (Polysciences) in 150 mM NaCl solution. The DNA-PEI mixture was vortexed for 10 seconds and incubated for 15 min at room temperature. Subsequently the mixture was added dropwise to the adherent HEK293T cells. One day after transfection, culture medium was refreshed with phenol red-free DMEM supplemented with 10% fetal bovine serum and 1% p/s. Another 24 hours later, the EV-containing culture supernatant was collected and centrifuged two times for 10 min at 300xg followed by two times at 10 min at 2000xg to remove dead cells and cell debris.

The supernatant of HEK293T cells transfected with Nluc-US28 (or separate EV and protein fractions isolated by SEC from this supernatant, see below) was added to white 96-wells plates. In the case of antagonist pre-treatment, a saturating concentration (100 nM) of the previously described monovalent US28 nanobody VUN100 in Hank’s Buffered Saline Solution (HBSS, Gibco, Thermo Fisher Scientific) supplemented with 0.05% (w/v) BSA was added to the wells and incubated with the supernatant for one hour at room temperature.^43^ Next, 10 μM of furimazine (NanoGlo Luciferase Assay System, Promega) in Hank’s Buffered Saline Solution (HBSS, Gibco, Thermo Fisher Scientific) supplemented with 0.05% BSA was added to the wells. The basal bioluminescence resonance energy transfer (BRET) ratios were measured using the PHERAstar plate reader (BMG Labtech) at 460-80 nm /620 nm-LP. Subsequently, increasing concentrations of CX_3_CL1-Alexa647, CCL5-Alexa647 and CXLC12-Alexa647 (Almac Group) in HBSS supplemented with 0.05% BSA were added to the wells and ligand binding was determined in kinetic mode for 1 hour using the PHERAstar plate reader at 460-80 nm /620 nm-LP.

#### Size exclusion chromatography

A SEC column was made by stacking Sepharose CL/2B (GE Healthcare, 17-0140-01) in PBS up to a 10ml column bead volume. Following centrifugation steps to remove dead cells and cell debris (2 × 10 min centrifugation at 300xg and 2 × 10 min at 2000xg), culture supernatant from Nluc-US28-transfected HEK293T cells was applied to the SEC column and allowed to enter the column by gravity. Collection of 0.5 ml fractions was started immediately and the EV-enriched fractions and protein-enriched fractions were used in a fluorescently labeled chemokine binding assay.

#### Mini-Gα_i_ recruitment assay

To collect HA-US28-expressing EVs, 1 million iHA-US28 U251 cells were seeded into 10cm^2^ cell culture dishes. The following day, receptor expression was induced with 1 μg/ml doxycycline (D9891, Sigma-Aldrich) in phenol red-free medium supplemented with 10% fetal bovine serum and 1% p/s. For control EVs, phenol red-free medium without doxycycline was added to the cells. After 48 hours, the culture medium was collected and centrifuged two times for 10 min at 300xg followed by two times for 10 min at 2000xg. The EV-containing supernatant was collected and concentrated using Centricon® Plus-70 (Millipore, Merck) according to manufacturer’s instructions.

HEK293T cells were transfected with 30 ng of pcDEF3-3xHA-CX_3_CR1-Nluc, 150 ng of pcDEF3-Venus-mini-Gαi, supplemented with empty pcDEF3 vector to a total of 2 μg DNA and 12 μg 25 kDa linear PEI in 150mM NaCl solution per 1 million cells. The DNA-PEI mixture was vortexed for 10 seconds and incubated for 15 min at room temperature. HEK293T cells were detached with Trypsin-EDTA (Gibco, Thermo Fisher Scientific) and resuspended in DMEM (Thermo Fischer). The HEK293T cell suspension was added to DNA-PEI mixture and cells were seeded in a poly-L-lysine-coated white 96-wells plate. Two days-post transfection, concentrated U251 iHA-US28 and control supernatant were pre-incubated with CX_3_CL1 (1 nM) and US28 antagonist VUN100 (31.6 nM) for 30 minutes at room temperature. Next, cell medium was aspirated and cells a final concentration of 10 μM of furimazine in HBSS supplemented with 0.05% BSA was added to the cells. The basal BRET ratio was measured using the PHERAstar plate reader at 475/30 nm and 535/30 nm. Subsequently, the pre-incubated supernatants were added to the cells and after 10 minutes incubation the ligand-induced BRET ratios were measured using the PHERAstar plate reader at 475/30 nm and 535/30 nm. For each condition, the increase in BRET upon CX_3_CL1 stimulation was presented as a percentage of the increase in BRET upon CX_3_CL1 in the absence of EVs (depicted as 100% CX3CR1 activation).

#### Schematics

Schematics were created using BioRender.com

### Quantification and statistical analysis

Data was analyzed and visualized using Prism 9.0 software (GraphPad). Statistical details of the experiments, including sample number, meaning of error bars and statistical tests used, are indicated in the figure legends. Statistical significance is denoted by asterisks with ∗p < 0.05 and ∗∗p < 0.01.

## Data Availability

• Data reported in this paper will be shared by the lead contact upon request • This paper does not report original code. • Any additional information required to reanalyze the data reported in this paper is available from the lead contact upon request.

## Supplemental information

Documen S1. Figure S1–S2
