# Supplementary material for: Exosomal release of the virus-encoded chemokine receptor US28 contributes to chemokine scavenging
Source: iScience. 2023 Jul 18;26(8):107412. doi: 10.1016/j.isci.2023.107412 (PMC10415803; doi:10.1016/j.isci.2023.107412)
Supplement: Documen S1. Figure S1–S2 [file mmc1.pdf]

iScience, Volume *26*

Supplemental information

Exosomal release of the virus-encoded chemokine receptor US28 contributes to chemokine scavenging

Maarten P. Bebelman, Irfan M. Setiawan, Nick D. Bergkamp, Jeffrey R. van Senten, Caitrin Crudden, Jan Paul M. Bebelman, Frederik J. Verweij, Guillaume van Niel, Marco Siderius, D. Michiel Pegtel, and Martine J. Smit

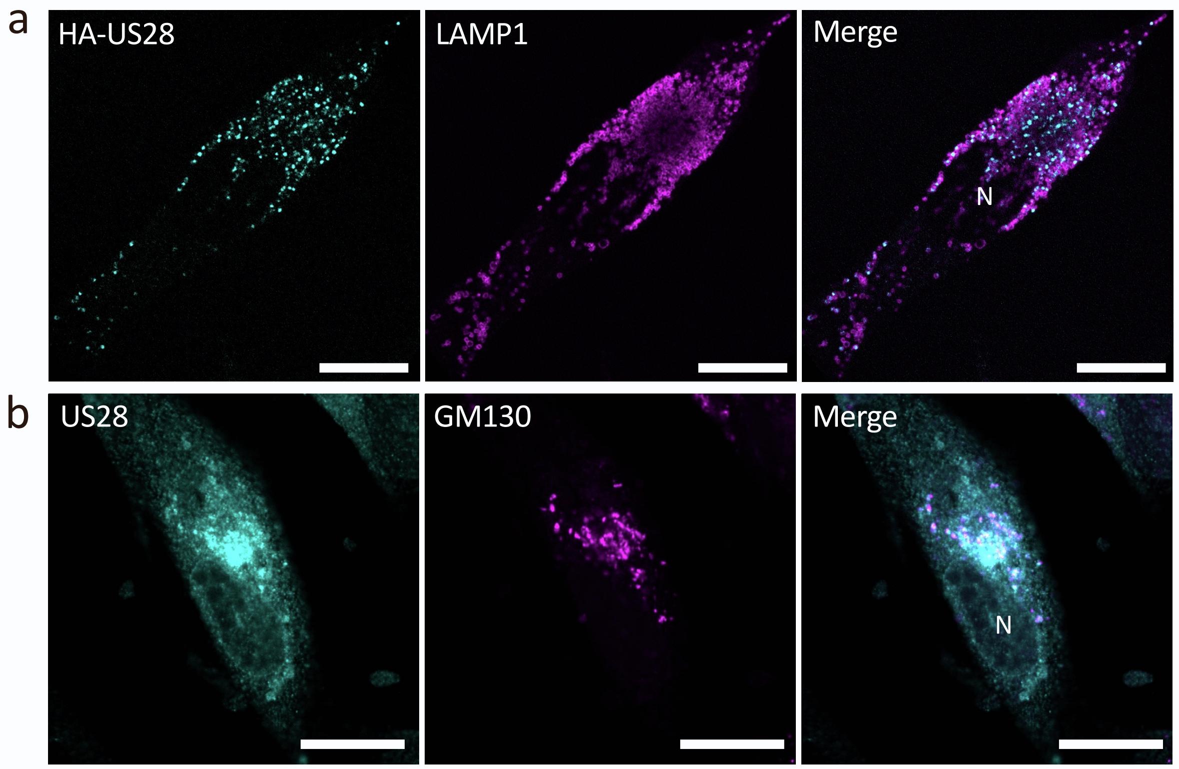

Figure S1. US28 localizes to late endosomes and the Golgi, Related to Figure 1 a) Localization of HA-US28 (anti-HA staining) and the late endosomal/lysosomal marker LAMP1 in doxycycline-induced iHA-US28-U251 cells. Scale bar 20 µm. N, nucleus. b) Localization of US28 (polyclonal anti-US28 antibody) and GM130 in HCMV Merlin-infected U251 cells 6 days post infection. Scale bar 20 µm. N, nucleus.

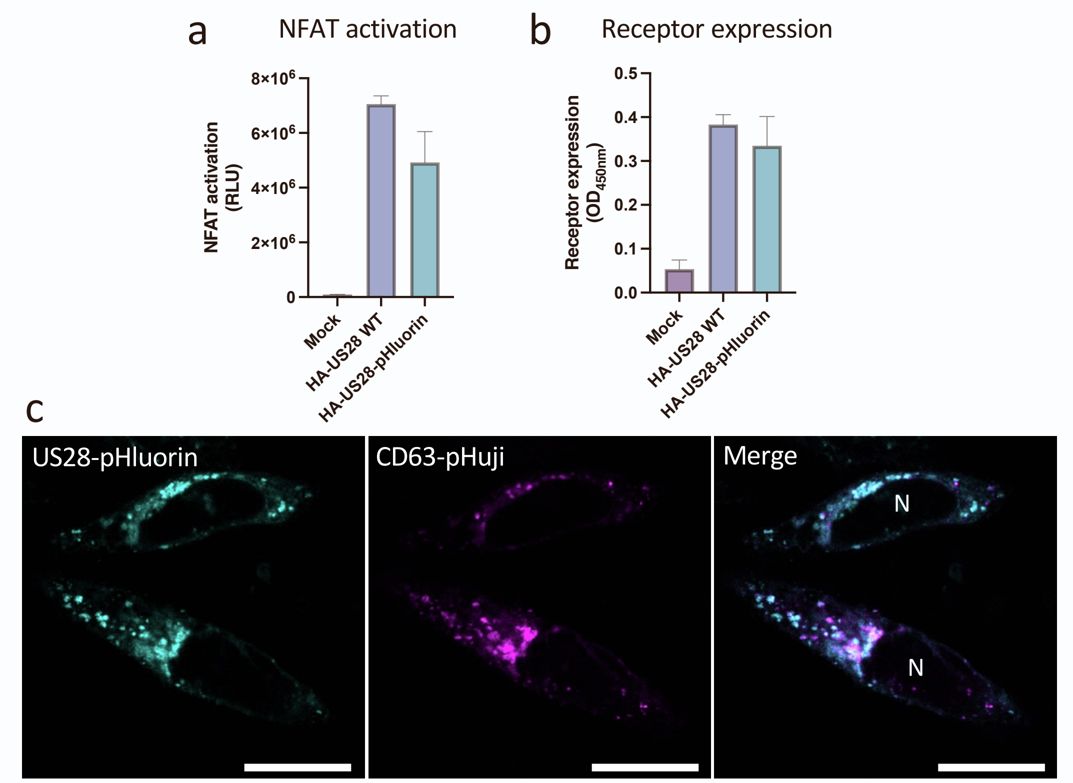

Figure S2. US28-pHluorin is functional and localizes to MVBs, Related to Figure 2 a) The effect of HA-US28 wildtype (WT) and HA-US28-pHluorin expression on NFAT activity in HEK293T cells transiently transfected with a NFAT reporter gene. Cells were transfected with equal amounts of receptor plasmid DNA. Graph depicts the mean ± SEM of three independent experiments with three replicates per experiment. b) Receptor expression levels in the NFAT reporter gene assay as determined by ELISA. Graph depicts the mean ± SEM of three independent experiments with three replicates per experiment. c) Localization of US28-pHluorin and CD63-pHuji in transiently transfected HeLa cells. Scale bar 20 µm.
